# How to tackle non-specific low back pain among adult patients? A systematic review with a meta-analysis to compare four interventions

**DOI:** 10.1186/s13018-023-04392-2

**Published:** 2024-01-03

**Authors:** Yawen Jiang, Yaping Xu, Xiangrui Kong, En Zhao, Chunxia Ma, Yihang Lv, Hongqi Xu, He Sun, Xiaojuan Gao

**Affiliations:** 1 Key Laboratory of Exercise and Health Sciences of Ministry of Education, Shanghai University of Sport, Shanghai, China; 2 School of Physical Education (Main Campus), Zhengzhou University, Zhengzhou, China; 3 Department of Public Physical and Art Education, Zhejiang University, Hangzhou, China; 4 Research Center of Sports and Health Science, School of Sports Science and Physical Education, Northeast Normal University, Changchun, China; 5 School of physical education, Henan university, Zhengzhou, China; 6 Synergetic Innovation Center of Kinesis and Health, School of Physical Education (Main Campus), Zhengzhou University, Zhengzhou, China

**Keywords:** Motor control training, Non-specific low back pain, Pilates, McKenzie, Physical therapy

## Abstract

**Objective:**

To tackle non-specific low back pain (NSLBP) among patients and find the most effective solution and to quantitatively synthesize the overall effect of motor control training (MCT) compared with Pilates, McKenzie method, and physical therapy (PT) in pain and physical function.

**Methods:**

Randomized controlled trials (RCTs) of four types of intervention (MCT, Pilates, McKenzie method, and PT) for LBP were collected by searching PubMed, Web of Science, EBSCOhost (Cochrane Central Register of Controlled Trials), and Scopus databases from the establishment of the database to September 30, 2023. The risk of bias was evaluated for included studies using the Revised Cochrane Risk of Bias tool for randomized trials (RoB 2.0). Taking pain and physical function in the experimental and control groups as outcome indicators, subgroup analysis was performed according to the intervention method to calculate the standardized mean difference (SMD) and 95% confidence interval (CI).

**Results:**

A total of 25 RCTs, including 1253 patients, were included. Meta-analysis showed that MCT effectively relieved pain [SMD = −0.65, 95% CI (− 1.00, − 0.29), *p* < 0.01] and improved physical function [SMD = −0.76, 95% CI (− 1.22, − 0.31), *p* < 0.01] comparing with other 3 types of intervention. Subgroup analysis suggested that MCT could alleviate pain [SMD = −0.92, 95% CI (− 1.34, − 0.50), *p* < 0.01] and improve physical function [SMD = −1.15, 95% CI (− 1.72, − 0.57), *p* < 0.01] compared with PT, but it had no statistical significance compared with Pilates [pain: SMD = 0.13, 95% CI (− 0.56, 0.83), *p* = 0.71; physical function: SMD = 0.10, 95% CI (− 0.72, 0.91), *p* = 0.81] and the McKenzie method [pain: SMD = −0.03, 95% CI (− 0.75, 0.68), *p* = 0.93; physical function: SMD = −0.03, 95% CI (− 1.00, 0.94), *p* = 0.95].

**Conclusions:**

MCT can effectively relieve pain and improve physical function in patients with NSLBP. It is more effective compared with PT for LBP, while no differences were detected between MCT and Pilates, as well as McKenzie method. Therefore, MCT, Pilates, and the McKenzie method should be encouraged as exercise interventions for NSLBP rehabilitation.

**Supplementary Information:**

The online version contains supplementary material available at 10.1186/s13018-023-04392-2.

## Introduction

Low back pain (LBP) is characterised as discomfort that occurs within the anatomical constraints defined by the costal margin superiorly and the inferior gluteal folds inferiorly, which may be accompanied by radicular leg pain [1, 2]. The majority of LBP cases are classed as non-specific low back pain (NSLBP), a state in which the particular pathoanatomical origin of the pain is unknown, accounting for more than 90% of all low back pain incidences [3]. As a serious public health problem, this disorder is a leading cause of long-term disability. Even, a well-done study involving 195 countries showed that LBP leads to a global decline in productivity [4, 5], resulting in direct and indirect costs that impose a huge economic burden on individuals and society [6, 7]. Many conservative treatments have been employed for pain management in LBP, such as pharmacological, acupuncture, and intra-articular injections [8–11]. But LBP is non-specific in most cases, and no pathoanatomical cause can be found. In such patients, management aims to reduce symptoms and disability, allowing the return to daily life activities and participation in physiotherapy. Therefore, much scientific interest raised to examine the countering effect of intervention on NSLBP [12–15].

Muscle structure influences muscle function, function influences structure, and pain/injury influences both, as is common in back muscles with LBP. In the short term, in addition to injury-related afferent input, acute pain and nociceptive stimulation can also affect back muscle function. In the long term, the persistent effects of pain and inflammatory mechanisms have additional effects on back muscle structure (e.g. atrophy, muscle fibre change, fatty infiltration, reduced strength/endurance) and function [16, 17]. In contrast, changes in back muscle function are considered to underlie the development and recurrence of low back pain [18]. These complex bidirectional interrelationships could drive cyclic processes in persistent or recurrent LBP.

Among the potential interventions used to counter NSLBP, motor control training (MCT) has received much attention as a neuromuscular rehabilitation method for relieving pain and improving physical function in patients with NSLBP. True to the complexity of motor control, MCT encompasses many aspects. It considers sensory and motor aspects of spine function, and each individual’s management program is tailored to features considered to be “suboptimal” on assessment. The basic premise of MCT is that, for many individuals, inputs from the spine and/or related tissues (including nociceptive) contribute to maintenance of symptoms secondary to “suboptimal” loading by person-specific features of alignment, movement, and muscle activation. MCT aims to identify and modify the sub-optimal features of motor control, with integration into function [19]. MCT is a type of exercise that aims to target these deep trunk muscles and improve spinal stability and posture. For example, an MCT programme includes low-level sustained isometric contraction of the deeper muscles of the trunk such as multifidus, transversus abdominis and pelvic floor muscles that are typically affected in the presence of pain [20]. The intervention focuses on the correction of motor control “faults”, such as optimisation of muscle activation or optimisation of posture and movement to modify loading of the lumbar spine [19, 21, 22].

In addition, Pilates [15, 23], the McKenzie method [24], and physical therapy [25] were also investigated as the protentional interventions to improve NSLBP. Pilates is a specific type of exercise therapy used to treat NSLBP. Pilates involves six fundamental principles: breathing, centring, concentration, control, precision, and flow [26]. Pilates shows effectiveness in patients with chronic non-specific low back pain (CNSLBP) to attenuate disability and pain [15]. To contrast, McKenzie intervention specifically has classified patients into 3 mechanical subgroups (derangement, dysfunction, or postural syndrome), by which to direct treatment [27]. Notably, Lam et al. (2018) compared McKenzie with other rehabilitation interventions. The result showed that there is moderate-to high-quality evidence that McKenzie method is superior to other rehabilitation interventions for reducing pain and disability [28, 29]. Finally, physical therapy (PT), comprising passive physical therapy, such as manipulation, chiropractic, osteopathy, massage, ultrasound, and electrical stimulation, is the most common evidence-based, reimbursable, and non-pharmacologic physician referral for NSLBP [30–32].

Among these interventions, MCT is particularly important since it directly targets the muscles responsible for spinal and pelvic stability [33–35]. Previous reviews have suggested that four interventions are all effective in relieving pain and improving physical function in patients with NSLBP [29, 36]. However, in the extracted studies, they only compare the counteractive effect with non-exercise intervention groups (passive control). In other words, MCT was not directly compared with Pilates, the McKenzie method, or PT for intervention effects, ignoring the possible effect of different intervention methods in the control group on the results.

Moreover, many investigations show that MCT is superior to Pilates [37], McKenzie method [36, 38], and PT [39] regarding the counteractive effect of NSLBP. However, the opposite results also exist [24, 40–43]. Since there have not been any reviews published to directly compare the effect of MCT with Pilates, the McKenzie method, and PT for NSLBP. The intervention effects of various treatment options, when MCT is compared against three others interventions, are not well known.

Therefore, this work investigates whether MCT on NSLBP can effectively reduce pain and improve physical function and aims to identify which interventions show the highest efficacy. On this basis, Pilates, the McKenzie method, and PT were included as the control group in this study. The effect of MCT on NSLBP and the difference with other interventions were investigated by directly comparing MCT with other interventions through the current review.

## Methods

This review was conducted in accordance with Preferred Reporting Items for Systematic Reviews and Meta-Analyses (PRISMA) [44] and was registered with OSF (osf.io/me2sq).

### Search strategy

The PubMed, Web of Science, EBSCOhost (Cochrane Central Register of Controlled Trials), and Scopus databases were searched for randomized controlled trials (RCTs) of the effect of motor control training on NSLBP to compare it with the effect of Pilates, the McKenzie method, and PT. The search time was from the establishment of the database to September 30, 2023. In addition, references incorporated in the studies were supplemented as relevant literature. A combination of subject terms and text words was taken as the search strategy. The search terms include: low back pain, stabili*, and randomized controlled trial. The complete search strategy of all databases can be found in Table 1.

**Table 1:** PubMed search strategy

Database	Complete search strategy	Hits (30/9/2023)
PubMed	(((((((((((((((((((Low Back Pain[MeSH]) OR (low back pain)) OR (low back pains)) OR (lumbago)) OR (lower back pain)) OR (lower back pains)) OR (low back aches)) OR (low backache)) OR (low backaches)) OR (lumbar pain)) OR (lumbar degenerat*)) OR (backache)) OR (back disorders)) OR (sciatica)) OR (coccyx)) OR (coccy*)) OR (spondylosis)) AND (((((stabili*) OR (motor control)) OR (sensorimotor)) OR (neuromuscular)) OR (perturbation))) AND ((((Randomized Controlled Trial) OR (randomized controlled trial)) OR (random*))	523
Web of Science	TS = ((Low Back Pain OR low back pain OR low back pains OR lumbago OR lower back pain OR lower back pains OR low back aches OR low backache OR low backaches OR lumbar pain OR lumbar degenerat* OR backache OR back disorders OR sciatica OR coccyx OR coccy* OR spondylosis) AND (stabili* OR sensorimotor OR motor control OR neuromuscular OR perturbation) AND (Randomized Controlled Trial OR randomized controlled trial OR random allocation OR random*))	1241
Scopus	((Low Back Pain[MeSH]) OR (lumbago) OR (lumbar pain) OR (backache) OR (sciatica)) AND ((stabili*) OR (sensorimotor) OR (motor control) OR (neuromuscular) OR (perturbation)) AND ((Randomized Controlled Trial[MeSH]) OR (randomized controlled trial) OR (random allocation) OR (random*))	1554
EBSCOhost	(“Low Back Pain”) OR (“low back pain”) OR (lumbago) OR (“lower back pain”) OR (“low back aches”) OR (“low backache”) OR (“lumbar pain”) OR (backache) OR (“back disorders”) OR (sciatica) OR (coccy*) OR (spondylosis) AND (stabili*) OR (sensorimotor) OR (“motor control”) OR (neuromuscular) OR (perturbation) AND (“Randomized Controlled Trial”) OR (“randomized controlled trial”) OR (random*)	812

### Eligibility criteria

The PICOS (Participants, Interventions, Comparisons, Outcomes and Study design) principle of evidence-based medicine was used as inclusion criteria for the literature [44] (Table 2).

**Table 2:** Eligibility criteria based on PICOS

PICOS	Criteria
Participation	Adults patients with NSLBP
Intervention	Motor control training
Comparison	MCT vs Pilates, McKenzie method, and PT
Outcome	Pain and physical function
Study design	Randomized controlled trials

*MCT* motor control training, *PT* physical therapy

The population group of interest were adults patients (≥ 18 years) with non-specific (no known pathoanatomical cause) low back pain (located below the costal margin and above the inferior gluteal folds, with or without leg pain) [2]. Therefore, study was excluded when the pain caused by pregnancy, infection, tumours, osteoporosis, fractures, structural deformities (e.g. scoliosis), lumbar disc herniation, inflammatory diseases, radicular syndrome, or cauda equina syndrome. The intervention for the experimental group was MCT, while the interventions for the control group were Pilates, the McKenzie method, and PT. The specific interventions were determined according to the group names chosen by the authors and the definitions given in Table 3. Studies were required to include at least one of the following outcome measures: subjective pain intensity [e.g. visual analogue scale (VAS), numerical pain rating scale (NPRS)], subjective physical function [e.g. Oswestry Disability Index (ODI), Roland-Morris Disability Questionnaire (RMDQ)]. This study is included in RCTs published in English.

**Table 3:** Definition of interventions in the intervention and control groups

Interventions	Definitions
MCT	Therapeutic exercise to modify specific motor control features for a broad, multidimensional view incorporating psychosocial aspects of LBP, considers the potential relevance of both “upregulation” (i.e. increased/augmented activation) and “downregulation” (i.e. decreased/compromised activation) of muscles [19]
Pilates	Training that follows traditional Pilates principles: centring (i.e. tightening the 'powerhouse' (trunk muscles)), concentration (i.e. cognitive attention while performing the exercises), control (i.e. postural management while performing the exercises), precision (i.e. accuracy of exercise technique), flow (i.e. smooth transition of movements within the exercise sequence) and breathing in coordination with the exercises [45]
McKenzie	McKenzie method is the direction of a repeated movement and/or sustained position producing improvement in symptoms. Training that follows traditional McKenzie principles such as repetitive passive spinal movements and sustained postures in specific directions [46]
Physical therapy	Treatments include passive physical therapy, such as thrust or non-thrust joint mobilization, soft tissue mobilization, neural tissue mobilization, massage, ultrasound and electrical stimulation [13]

### Literature screening and data extraction

Two researchers independently screened the literature and extracted information before cross-checking. First, researchers read the title and abstract of the article to exclude obviously irrelevant studies. Then, they read the full text and screened it according to the inclusion and exclusion criteria. When disagreements appeared, a third researcher would be involved in the discussion of inclusion or not. The included articles and references of relevant systematic reviews were checked to ensure that all relevant articles were searched using the search strategy.

The content of data extraction mainly includes: 1) basic information of included studies: first author and year of publication; 2) basic information of the study subjects: sample size, age, sex, and duration of NSLBP; 3) information on interventions: type and duration; 4) outcome indicators: mean and standard deviation of pain and physical function after the intervention. Data were extracted by two independent assessors. Any discrepancies were discussed by two researcher with disagreements adjudicated by a third researcher.

### Risk of bias assessment and GRADE

The risk of bias was evaluated for included studies using The Revised Cochrane Risk of Bias tool for randomized trials (RoB 2.0). For each included study, two researchers independently performed a risk of bias assessment according to five domains with the results of “low risk of bias”, “high risk of bias” or “some concerns of bias”. Any disagreements were discussed and solved with a third reviewer. The Grading of Recommendations Assessment, Development and Evaluation (GRADE) approach was used to evaluate the overall quality of the evidence for each outcome, which ranges from high to very low quality and is based on five domains: limitations of design, inconsistency of results, indirectness, imprecision, and other factors, such as publication bias.

### Statistical analysis

Meta-analysis was performed using Review Manager 5.4. Pain and physical function scores were continuous variables. The extracted data from the MCT group were included in the experimental group of the meta-analysis, and the extracted data from the Pilate, McKenzie, and PT groups were included in the control group of the meta-analysis. Standardized mean difference (SMD) was used as the effect magnitude, and estimates and a 95% confidence interval (CI) were given for each effect size. A random effects model was used for all analyses. SMD effect sizes were calculated in Review Manager V.5.3 using Hedges’ g method. Clinical relevance was defined as small: SMD ≤ 0.5; moderate: SMD ranging from 0.5 to 0.8; large: SMD ≥ 0.8 [47]. When studies were reverse scaled (i.e. higher values indicated better outcomes rather than lower values), the mean in each group was multiplied by − 1 as recommended in the Cochrane Handbook [48]. The heterogeneity between the results of the included studies was analysed by the *χ*^2^ test, while its magnitude was determined quantitatively by combining *I*^2^. Homogeneity was considered good if *P* > 0.5 and *I*^2^ < 50%. High heterogeneity was considered to exist if *P* < 0.5 and *I*^2^ ≥ 50% [48]. If the heterogeneity was high, subgroup analysis was performed. Subgroup analysis was performed according to the intervention approach of the control group to identify whether relationships between exercise and outcomes differed depending on the type of control group. Additionally, the publication bias of meta-analysis was detected by Begg's test and funnel plot.

### Research results

#### Literature search process and results

**Fig. 1:**
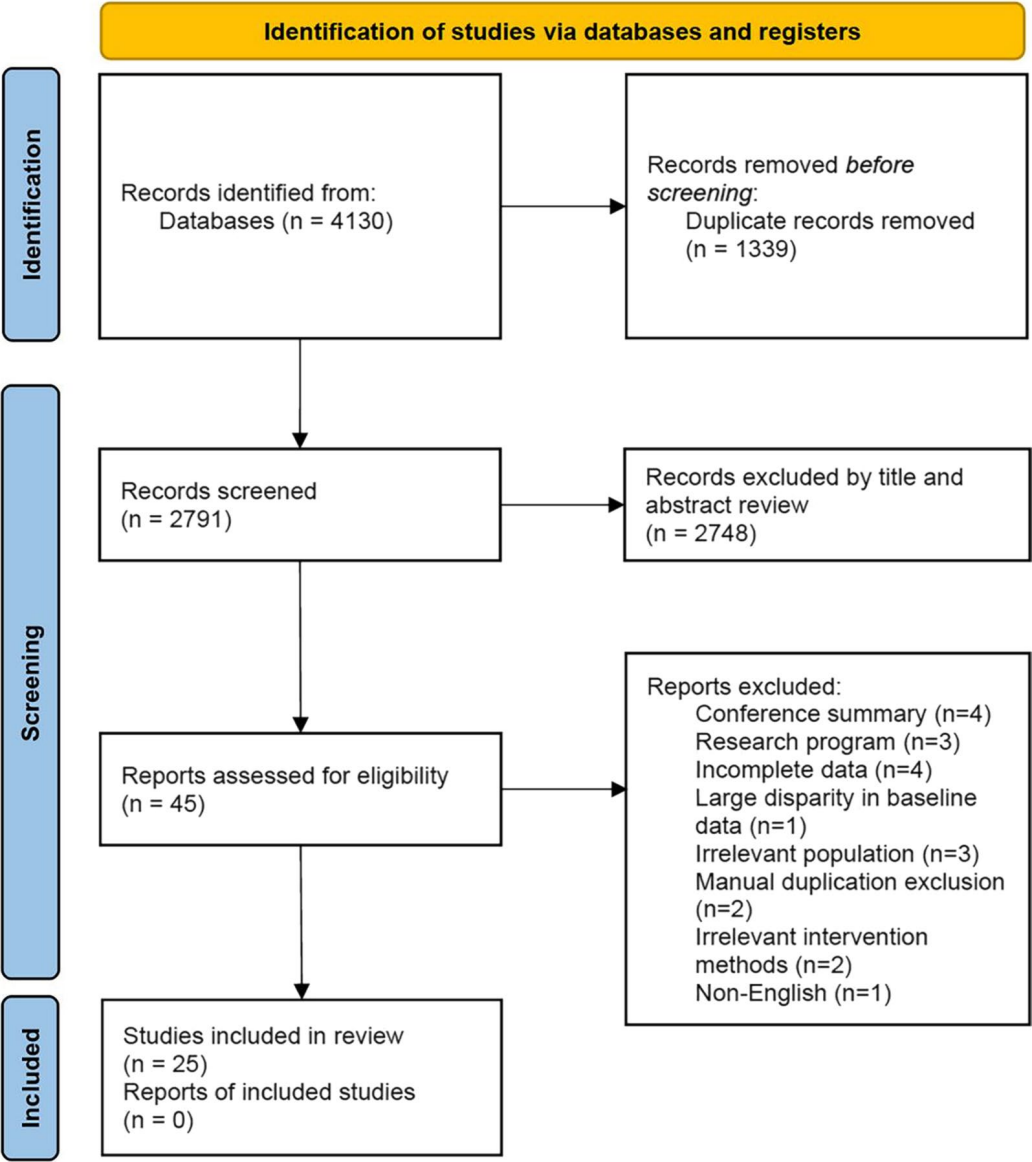
The literature search and inclusion process of this study

A total of 4130 articles related to the topic of this study were retrieved from four databases, and three articles were found through the references of published systematic reviews. Through the preliminary screening of titles and abstracts, 45 articles were obtained. After the researchers read the full text, 25 articles were eventually included in the meta-analysis. The flowchart in Fig. 1 depicts the detailed steps of the literature search using meta-analysis [37–42, 49–67].

### Basic characteristics of the included studies

Twenty-five studies were extracted. There were 1253 subjects with mixed sexes, aged 18–65 years. The sample size ranged from 12 to 154 subjects per study. In terms of intervention characteristics, the intervention duration was 3–24 weeks. Four articles compared MCT with Pilates [37, 40, 49, 61], three articles compared MCT with the McKenzie method [38, 42, 67], and eighteen articles compared the effects of MCT and PT [39, 41, 50–60, 62–66] (Table 4).

**Table 4:** Characteristics of studies included in meta-analysis

Study	N		Sex (males, n)		Age (years, M ± SD)		Mean baseline pain duration	Intervention type		Study duration	Outcome indicators	
	E	C	E	C	E	C		E	C		Pain	Physical function
Akodu et al. [37]	10	10	13	49.10 ± 11.85	45.30 ± 11.31	≥ 3 months	MCT	Pilates	4 weeks	NRS	RMDQ	
Bhadauria et al. [49]	12	12	6	11	32.75 ± 11.73	35.33 ± 12.88	> 3 months	MCT	Pilates	3 weeks	VAS	MODQ
Gagnon et al. [61]	6	6	NR	30.33 ± 12.40	36.00 ± 11.43	E: < 3 months16.7% ≥ 3 months83.3% C: < 3 months33.3% ≥ 3 months66.7%	MCT	Pilates	6 weeks	VAS	ODI	
Halliday et al. [67]	30	32	6	6	48.3 ± 14.2	48.8 ± 12.1	> 3 months	MCT	McKenzie	8 weeks	VAS	PSFS
Hosseinifar et al. [38]	15	15	NR	40.1 ± 10.8	36.6 ± 8.2	> 3 months	MCT	McKenzie	6 weeks	VAS	FRI	
Kofotolis et al. [40]	36	37	0	0	39.11 ± 8.68	41.22 ± 8.49	> 12 weeks	MCT	Pilates	8 weeks	SF-36	RMDO
Miller et al. [42]	15	14	8	7	54 ± 15	44 ± 16	> 12 weeks	MCT	McKenzie	6 weeks	VAS	FSQ
Bibi et al. [50]	30	30	28	30	29.80 ± 4.97	30.86 ± 6.06	NR	MCT	PT	4 weeks	VAS	MODI
Costa et al. [63]	77	77	32	29	54.6 ± 13.0	52.8 ± 12.7	≥ 3 months	MCT	PT	8 weeks	NRS	RMDQ
Critchley et al. [56]	72	71	21	29	44 ± 13	45 ± 12	> 12 weeks	MCT	PT	8 sessions	VAS	RDQ
Doğancalı et al. [52]	14	14	10	25.92 ± 3.26	27.64 ± 4.06	> 6 months	MCT + PT	PT	5 weeks	VAS	ODI	
Ge et al. [57]	15	16	0	64.60 ± 3.71	64.12 ± 2.96	> 3 months	MCT + PT	PT	4 weeks	VAS	ODI	
Kim et al. [60]	27	26	0	29.7 ± 3.9	28.6 ± 3.2	> 3 months	MCT	PT	8 weeks	VAS	-	
Salavati et al. [55]	20	20	20	20	32.60 ± 7.80	29.93 ± 5.18	> 12 months	MCT	PT	4 weeks	VAS	ODI
Shoukat et al. [62]	20	20	27	18–65	NR	MCT + PT	PT	4 months	VAS	MODQ		
Waseem et al. [64]	53	55	35	36	46.39 ± 7.43	45.50 ± 6.61	> 12 weeks	MCT	PT	6 weeks	-	ODI
You et al. [54]	20	20	8	11	50.35 ± 9.26	51.30 ± 7.01	> 6 months	MCT + PT	PT	8 weeks	VAS	ODI
Akhtar et al. [39]	53	55	NR	46.39 ± 7.43	45.50 ± 6.61	NR(CNSLBP)	MCT	PT	6 weeks	VAS	-	
Srivastav et al. [53]	15	15	NR	30–50	> 3 months	MCT	PT	6 weeks	NPRS	MODQ		
Hwang et al. [59]	7	7	4	4	45.71 ± 8.55	44.85 ± 7.92	> 12 weeks	MCT	PT	4 weeks	VAS	ODI
Wälti et al. [65]	14	13	5	8	41.57 ± 9.77	41.71 ± 12.21	> 3 months	MCT	PT	8–12 weeks	NRS	RMDQ
Ghorbanpour et al. [58]	15	15	7	7	23.8 ± 3.52	20.93 ± 1.22	> 6 months	MCT	PT	6 weeks	VAS	QBPDS
Bhatnagar et al. [51]	15	15	NR	38.86 ± 8.79	41.46 ± 8.61	> 3 months	MCT	PT	6 weeks	NPRS	ODI	
Bi et al. [66]	23	24	13	13	29.08 ± 2.68	30.87 ± 2.81	≥ 3 months	MCT	PT	24 weeks	VAS	ODI
Mohseni-Bandpei et al. [41]	10	10	0	34.71 ± 5.03	34.91 ± 6.29	NR(CNSLBP)	MCT + PT	PT	8 weeks	VAS	ODI	

*E* experimental group, *C* control group, *MCT* motor control training, *PT* physical therapy, *VAS* visual analogue scale, *NPRS* numerical pain rating scale, *SF-36* Short Form-36v2 Health Survey, *RMDQ* Roland-Morris Disability Questionnaire, *MODQ* Modified Oswestry Disability Questionnaire, *ODI* Oswestry Disability Index, *PSFS* Patient-Specific Functional Scale, *FRI* Functional Rating Index questionnaire, *FSQ* Functional Status Questionnaire, *QBPDS* Quebec Low Back Pain Disability Scale, *MODI* modified disability, *NR* not reported

### Risk of bias assessment

**Fig. 2:**
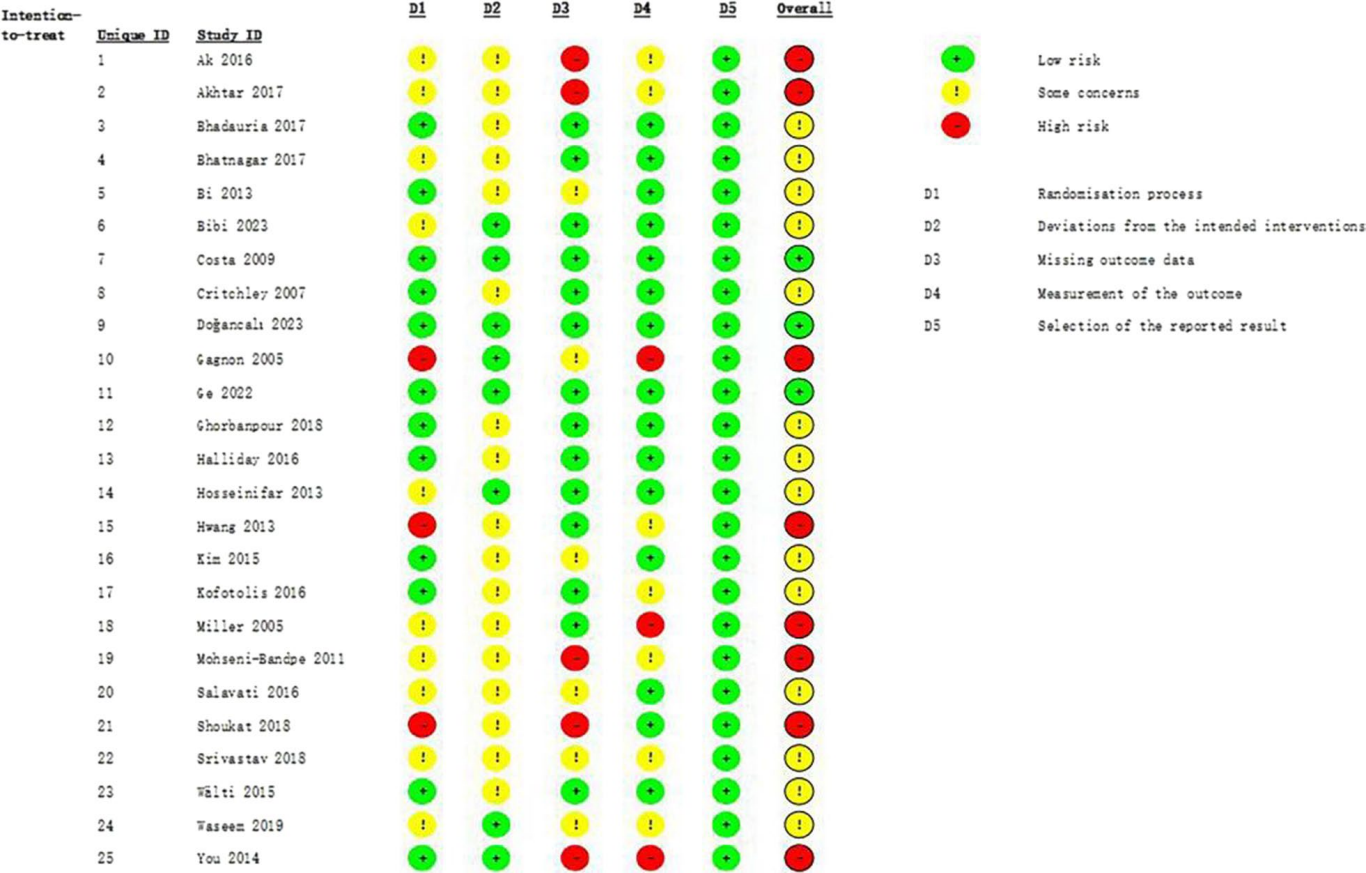
Risk of bias assessment for each study

**Fig. 3:**
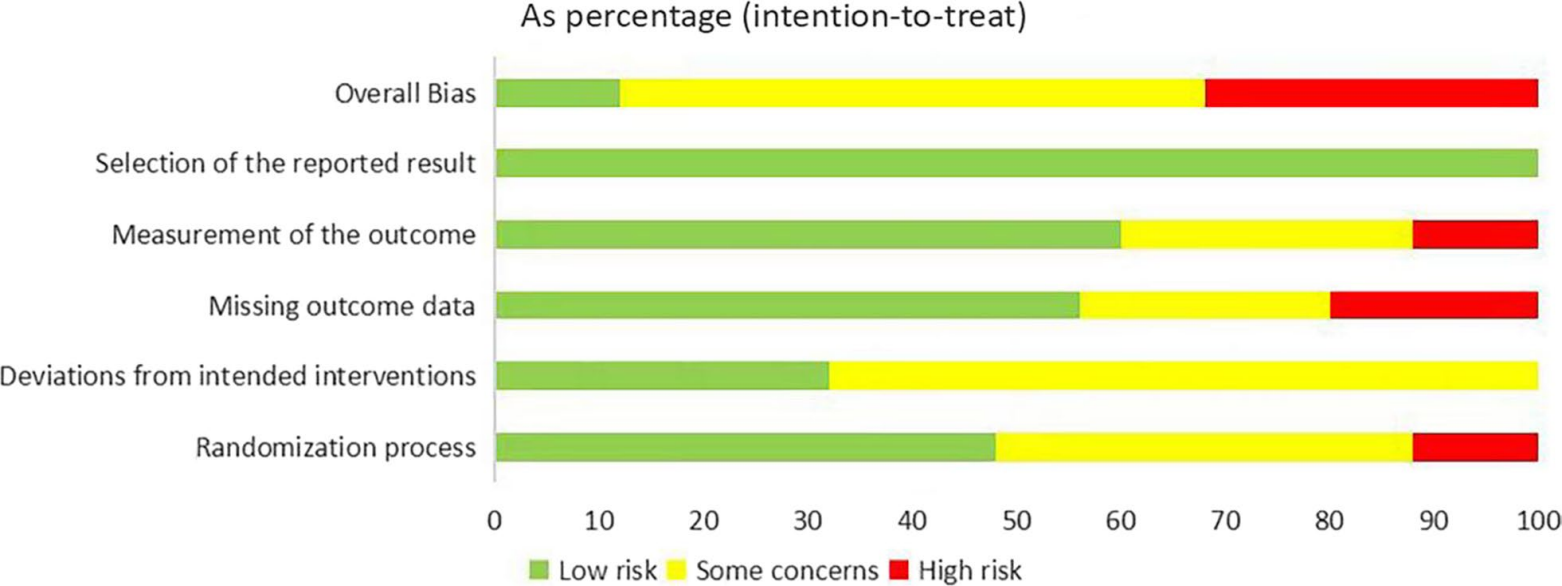
Overall risk of bias for included studies

The level of agreement between the three authors who assessed the risk of bias of the included articles was 93.3%. The majority of studies (56%; *n* = 14) were classified as "some concern", while eight studies (32%) were judged as having "some concern". Three studies were found to have a low risk of bias (12%). The main items that resulted in an overall high risk of bias in 14 of the 25 studies were improper handling of missing outcome data and inadequate blinding of participants and personnel. A summary of the risk of bias for each included study and each domain is given in Figs. 2 and 3.

### Meta-analysis results

#### Effects of MCT on pain in patients with NSLBP

Twenty-four included studies evaluated the effect of MCT on pain in patients with NSLBP. One of the included studies did not evaluated the effect of pain [64]. A total of 1135 subjects were enrolled, including 561 subjects in the experimental group and 574 subjects in the control group. Mainly, three tools were used for pain assessment, including visual analogue scale (VAS, 0–10 points), numerical pain rating scale (NPRS, 0–10 points), and Short Form-36v2 Health Survey (SF-36, 0–100 points).

**Fig. 4:**
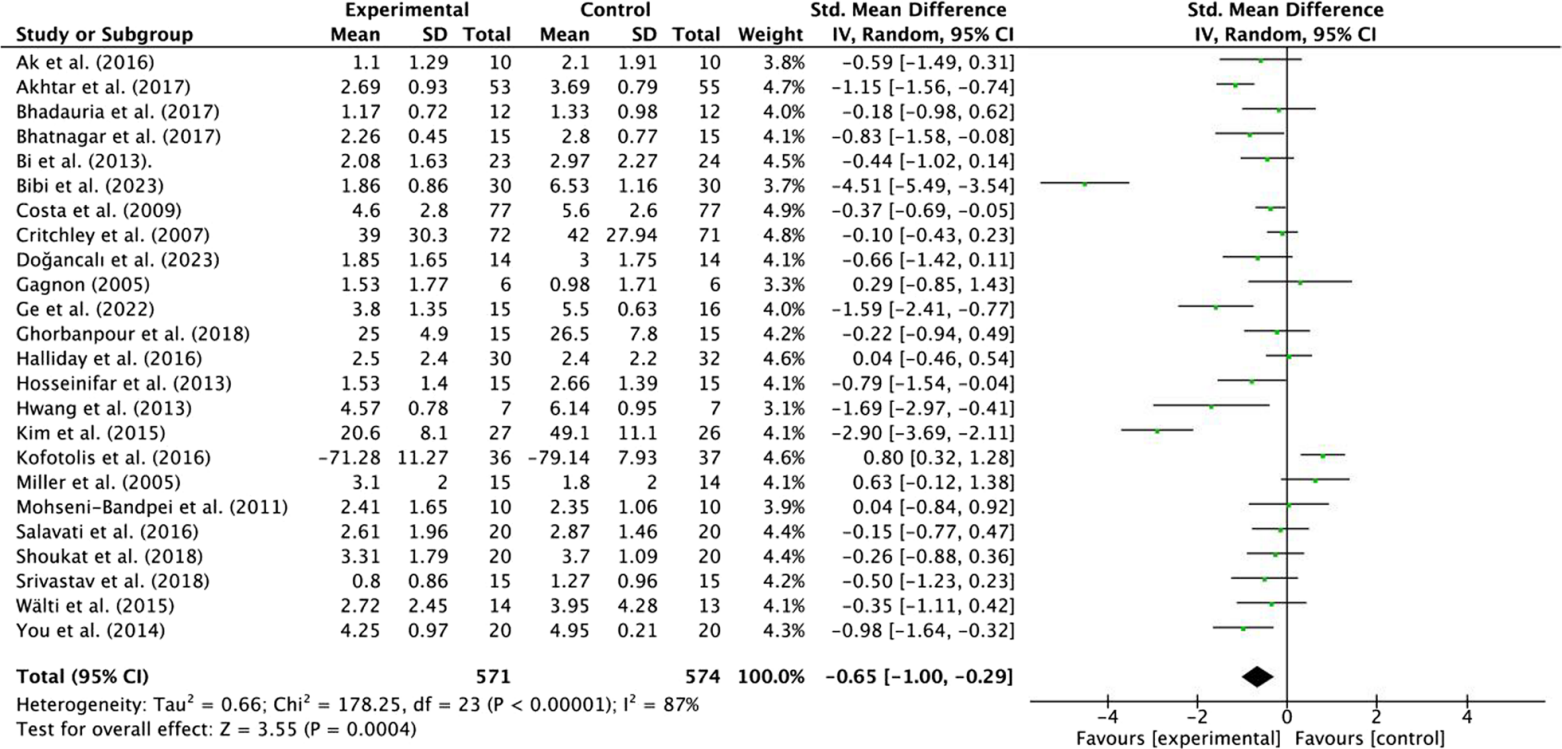
Effects of MCT on pain in patients with NSLBP

Moderate quality evidence suggested that MCT results in a moderate, statistically significant, and clinically better effect than other interventions (Pilates, McKenzie and PT) [SMD = −0.65, 95% CI (− 1.00, − 0.29), *p* < 0.01] (Fig. 4; Additional file 1: Table S1). Moreover, there was a high heterogeneity for the overall effect (Tau^2^ = 0.66, *P* < 0.01; *I*^2^ = 87%). The weight value of every study ranged from 3.3 to 4.9% in the analysis.

#### Effects of MCT on physical function in patients with NSLBP

Twenty-three included studies evaluated the effect of MCT on physical function in patients with NSLBP. Two included studies did not evaluated the effect of physical function [39, 60]. Among the 1092 subjects, there were 544 subjects in the experimental group and 548 subjects in the control group. Mainly, seven tools were used for physical function assessment, including Roland-Morris Disability Questionnaire (RMDQ), Modified Oswestry Disability Questionnaire (MODQ), Oswestry Disability Index (ODI), Patient-Specific Functional Scale (PSFS), Functional Rating Index (FRI) questionnaire, Functional Status Questionnaire (FSQ), and Quebec Low Back Pain Disability Scale (QBPDS).

**Fig. 5:**
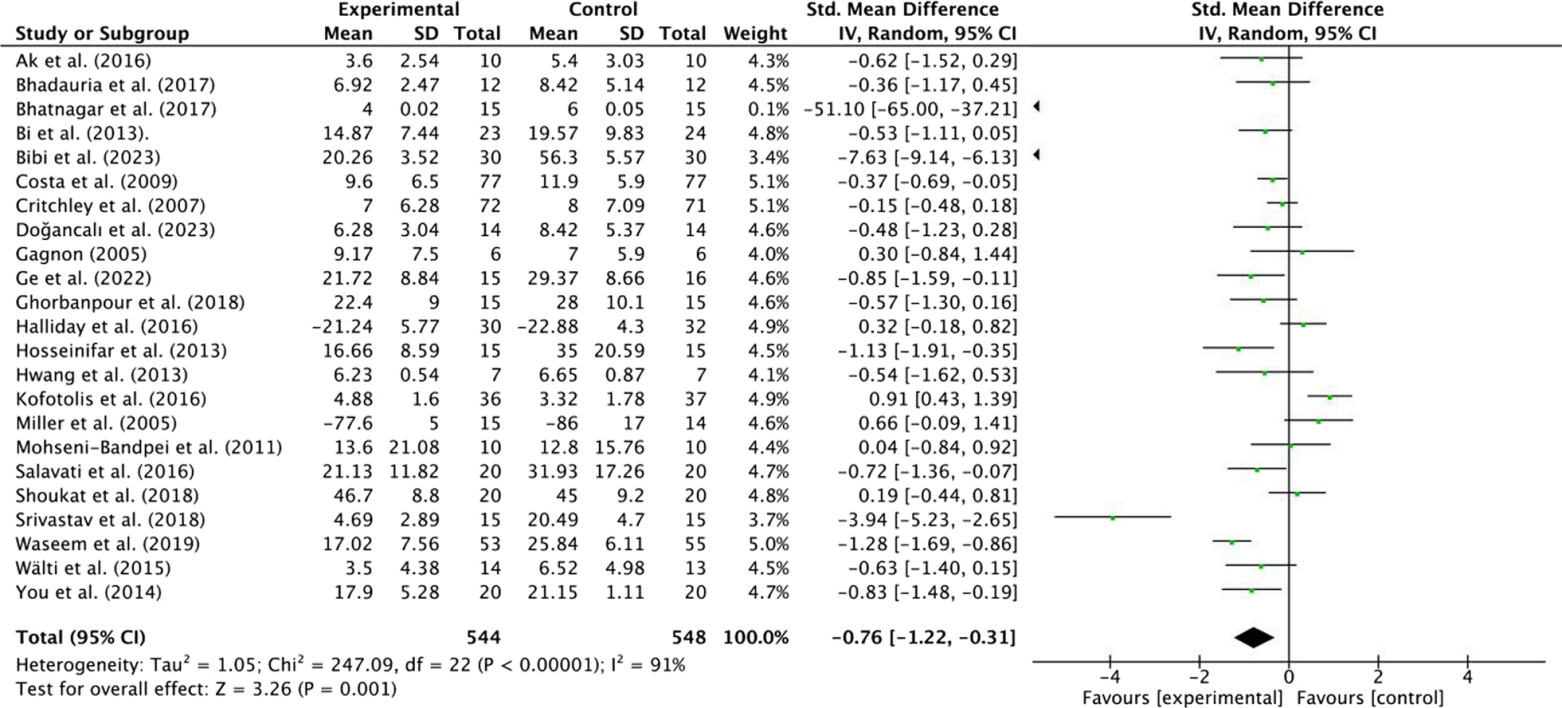
Effects of MCT on physical function in patients with NSLBP

Moderate quality evidence suggested that MCT results in a moderate, statistically significant, and clinically better effect than other interventions (Pilates, McKenzie, and PT) [SMD = −0.76, 95% CI (− 1.22, − 0.31), *p* < 0.01] (Fig. 5; Additional file 1: Table S1). Moreover, there was a high heterogeneity for the overall effect (Tau^2^ = 1.05, *P* < 0.01, *I*^2^ = 91%). The weight value of every study ranged from 0.1 to 5.1% in the analysis.

#### Subgroup analysis

**Fig. 6:**
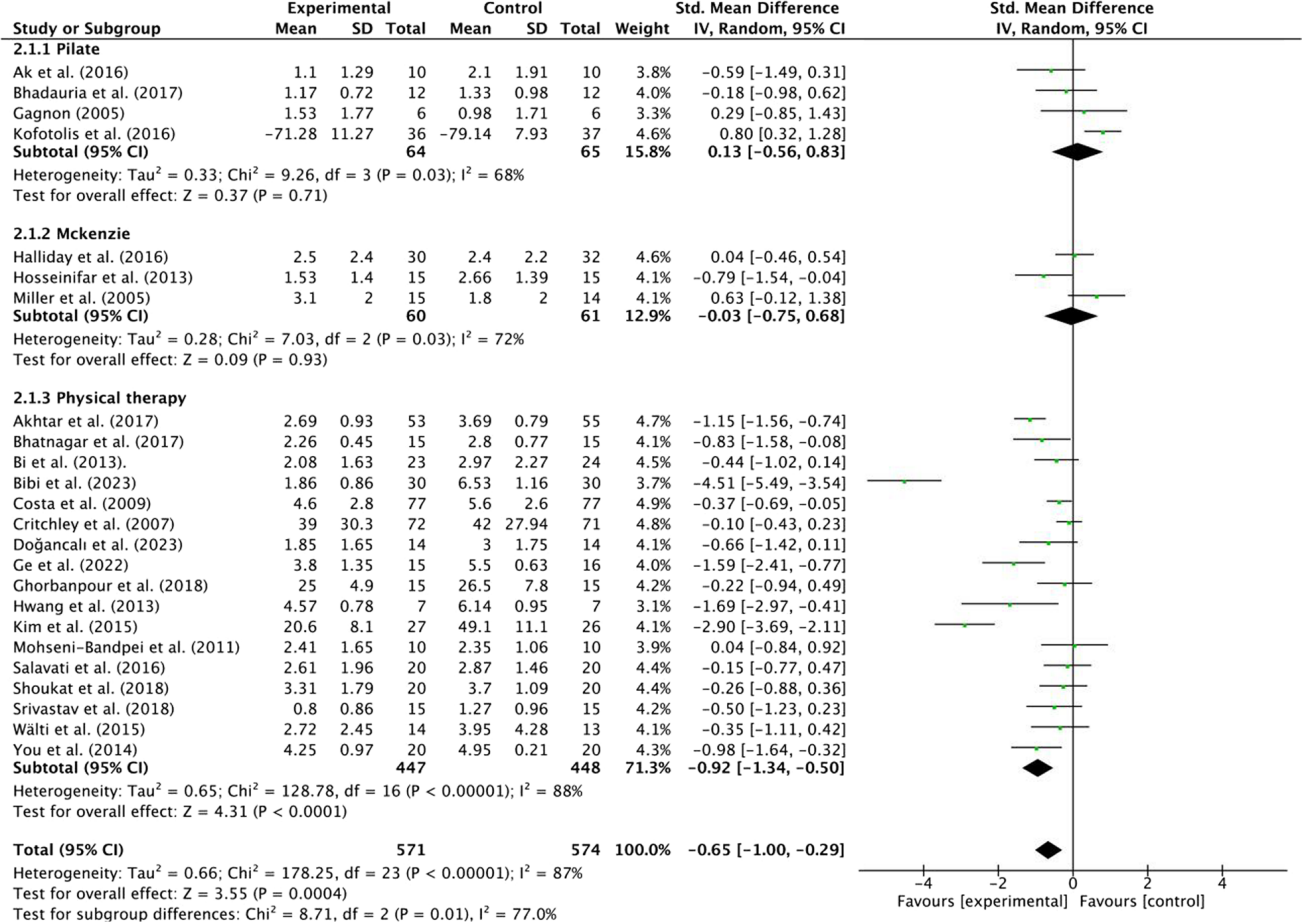
Forest plots of subgroup analysis of different interventions in the control group (pain)

*Pain* A subgroup analysis revealed an effect of the intervention method in the control group on the relationship between MCT and pain. Moderate quality evidence suggested that MCT results in a large, statistically significant, and clinically better effect than PT [SMD = −0.92, 95% CI (− 1.34, − 0.50), *p* < 0.01, Tau^2^ = 0.66, *I*^2^ = 87%]. Low-quality evidence suggested that MCT is not statistically better than Pilate [SMD = 0.13, 95% CI (− 0.56, 0.83), *p* = 0.71, Tau^2^ = 0.33, *I*^2^ = 68%] and McKenzie [SMD = −0.03, 95% CI (− 0.75, 0.68), *p* = 0.93, Tau^2^ = 0.28, *I*^2^ = 72%]. The size of the effect was also not clinically relevant (Fig. 6; Additional file1: Table S1).

**Fig. 7:**
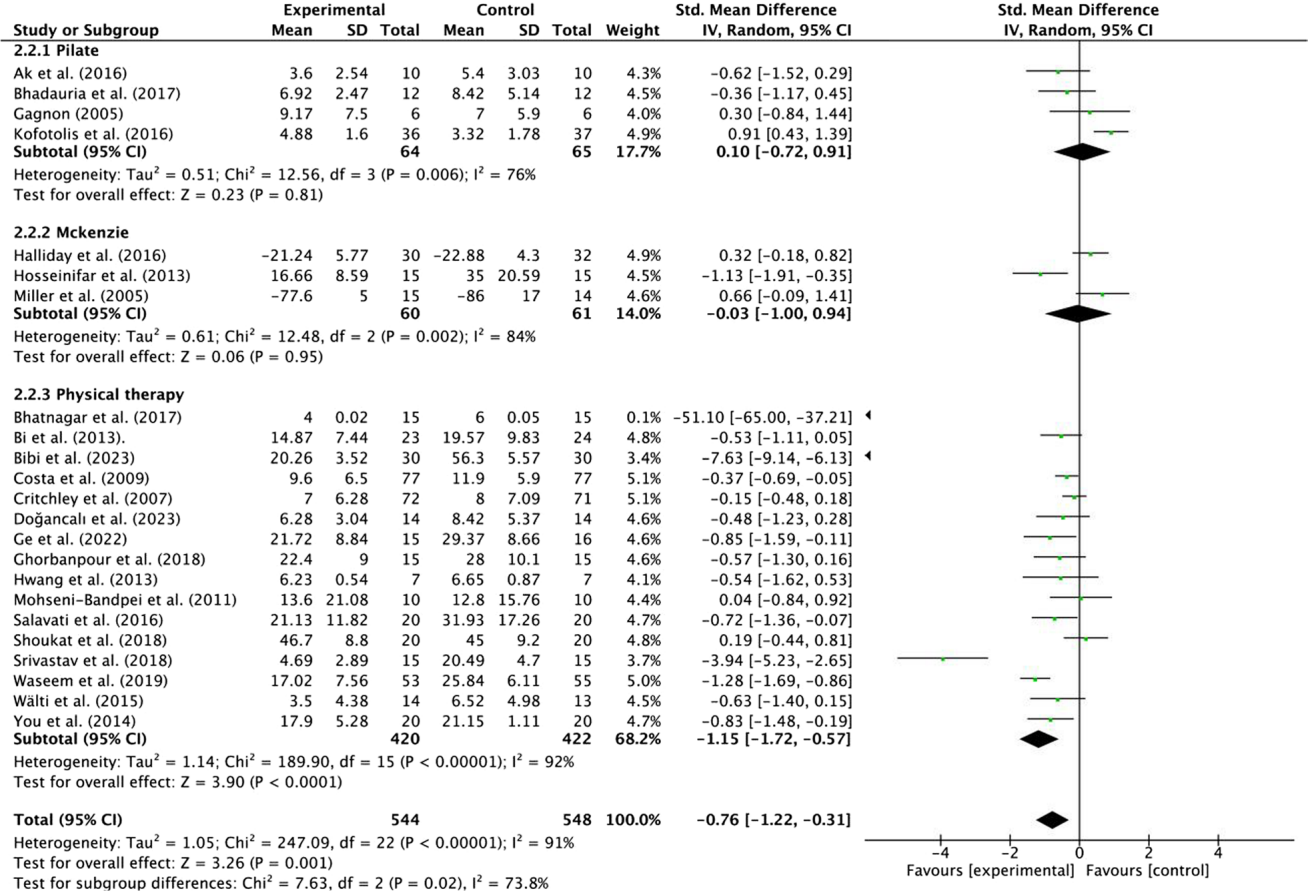
Forest plots of subgroup analysis of different interventions in the control group (physical function)

*Physical function* A subgroup analysis revealed an effect of the intervention method in the control group on the relationship between MCT and physical function. Moderate quality evidence suggested that MCT results in a large, statistically significant, and clinically better effect than PT [SMD = −1.15, 95% CI (− 1.72, − 0.57), *p* < 0.01, Tau^2^ = 1.14, *I*^2^ = 92%]. Low-quality evidence suggested that MCT is not statistically better than Pilate [SMD = 0.10, 95% CI (− 0.72, 0.91), *p* = 0.81, Tau^2^ = 0.51, *I*^2^ = 76%] and McKenzie [SMD = −0.03, 95% CI (− 1.00, 0.94), *p* = 0.95, Tau^2^ = 0.61, *I*^2^ = 84%]. The size of the effect was also not clinically relevant (Fig. 7; Additional file 1: Table S1).

#### Bias test

**Fig. 8:**
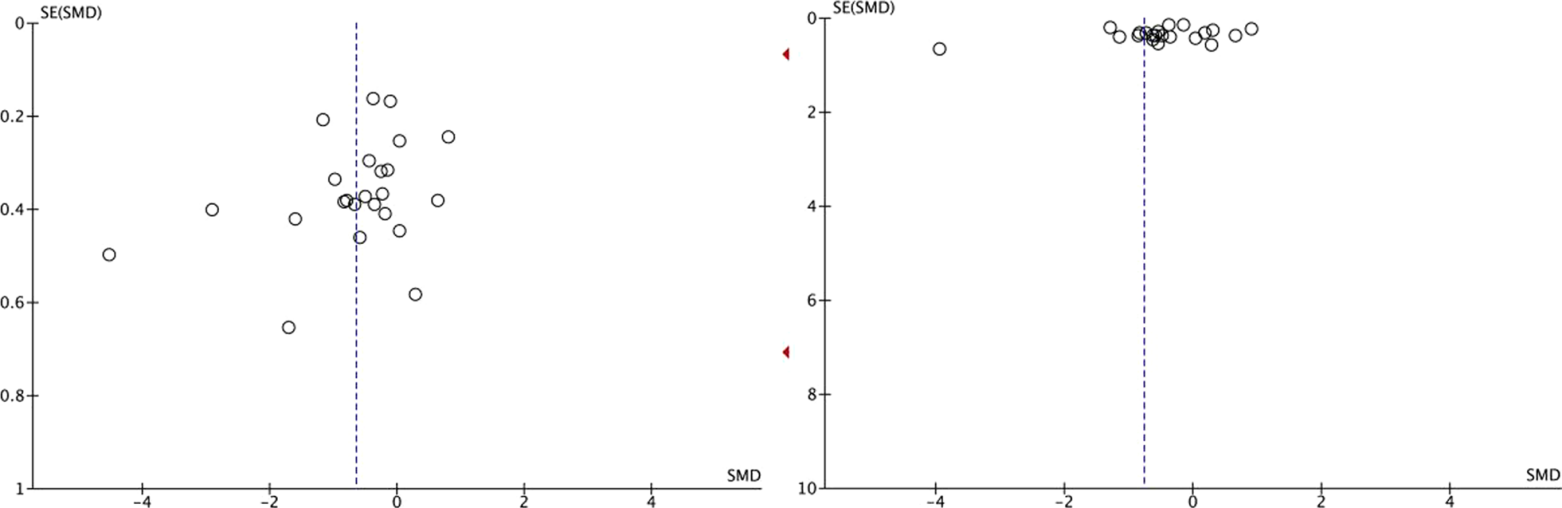
Funnel plot of the effect of MCT on patients with NSLBP

Publication bias tests were performed for the two meta-analyses of this study. Begg's (pain: *P* = 0.097; physical function: *P* = 0.051) linear regression tests showed their *P* > 0.05. In the funnel plot, there is no significant asymmetry in the scatter distribution. Therefore, there is no publication bias in both meta-analyses (Fig. 8).

## Discussions

This study is the first meta-analysis to directly compare MCT with Pilates, the McKenzie method, and PT and recommends the best exercise intervention approach. In this study, Pilates, McKenzie method, and PT were included as control groups, and the effect of MCT on NSLBP and the difference with other interventions were investigated by directly comparing MCT with other interventions.

### The intervention effect of MCT

This study reveals that MCT is effective in relieving pain and improving physical function in patients with NSLBP, which is consistent with the results of several previous studies [29, 36]. In the overall effect test, the pain effect size is negative in 19 studies, while the physical function effect size is negative in 17 studies, indicating that pain relief and physical function improvement can be achieved. The reason may be related to the activation, inhibition, and atrophy of the multifidus muscle. Symptoms in patients with NSLBP are mainly caused by altered muscle activation patterns and muscle atrophy. Studies have shown that activation of the multifidus muscle is suppressed in patients with acute NSLBP; activation of superficial muscle in the back is enhanced, and muscle fibre types are transformed in patients with subacute NSLBP; muscle structure is impaired (especially the multifidus muscle), and the proportion of type I muscle fibres is reduced in patients with chronic NSLBP. For the rehabilitation of patients with NSLBP, the activation mode needs to be considered first depending on individual adaptations, followed by resistance training to enhance strength and endurance [16, 68]. MCT can enhance joint stability, neuromuscular activation, and muscle strength and endurance, thus relieving pain and improving physical function [69]. For patients with NSLBP, activating the overinhibited multifidus muscles and restoring their cross-sectional areas can restore muscle health and relieve NSLBP.

### The intervention effect of MCT comparing with Pilates

The overall pooled effect size in this study is moderate [47], with *d* = −0.65 (pain) and *d* = −0.76 (physical function), respectively. Previous meta-analyses have also shown that MCT has a smaller effect size than other interventions for NSLBP [70]. We speculate that the reason is related to the different intervention methods of the control group. Therefore, to explore whether this is the reason for the small pooled effect size, a subgroup analysis was conducted in this study.

The subgroup analysis shows that there is no statistically significant difference in pain reduction and physical function improvement in patients with NSLBP with MCT compared to Pilates. The results suggest that MCT is similarly effective to Pilates for NSLBP and explain why the overall pooled effect size is small. The powerhouse is a central concept within Pilates and includes mainly the isometric contraction of deep muscles (i.e. multifidus, transversus, pelvic floor, and diaphragm), which can activate the deep muscles of the back and improve the muscle function of LBP [71]. The mechanism of Pilates for the treatment of LBP is similar to that of MCT. Thus, approaches specific to muscle activation and recovery, such as MCT and Pilates, are needed for the rehabilitation of LBP patients [16]. And previous studies have also demonstrated that the most beneficial programs for NSLBP included Pilates and MCT [33].

### The intervention effect of MCT comparing with McKenzie

As with Pilates, the McKenzie method is similar to MCT in reducing pain and improving physical function in patients with NSLBP. Directional preference, also known as the "derangement", is the most common subgroup of the McKenzie method and is associated with a rapid improvement in symptoms as a result of performing a "directional-preference" exercise. The directional preference of a patient is the direction in which a repeated movement and/or sustained position produces an improvement in symptoms. Centralization is a phenomenon in which symptoms down the lower extremity are progressively abolished in a distal to proximal direction. The presence of centralization is associated with good prognosis in patients with LBP [72]. Despite the difference in theoretical rationale for how MCT and McKenzie method might help people with chronic LBP there is limited evidence that TrA thickness can be increased after MCT and McKenzie method [38, 67]. A recent study showed that these two treatment methods both have a comparable effect on improving physical function. Therefore, both are equally effective in patients with NSLBP [24].

### The intervention effect of MCT comparing with PT

However, compared with PT, MCT achieves a large effect size for pain (*d* = −0.92) and physical function (*d* = −1.15) in patients with NSLBP, indicating that MCT is superior to PT interventions for NSLBP patients. The reason may be related to the treatment methods of PT. PT includes manipulation, chiropractic, osteopathy, massage, ultrasound, and electrical stimulation and is not sufficient to improve joint stability, neuromuscular activation, and muscle strength and endurance. Previous studies have also demonstrated that MCT was effective in pain relief compared to PT [29, 36].

In this study, a subgroup analysis based on the intervention method of the control group proved that there was no difference in the intervention effect of MCT, Pilates, and the McKenzie method, suggesting that all three interventions can be effective in the treatment of NSLBP. In addition, since psychosocial factors may be critical in individual interventions and interact with physical characteristics (including pain and physical function) [73], they need to be considered in the comprehensive management of NSLBP. Therefore, an appropriate method of rehabilitation can be selected from these three exercises according to the needs of patients with NSLBP and the ability of physicians to enhance individual compliance, thus achieving better intervention effects.

### Limitation of this study

As a meta-analysis, this study is limited by some uncontrollable factors. (1) Only published English literature was included in this study, and the included articles may be not comprehensive due to copyright and other factors. A more extensive search should be conducted in the future. (2) Due to the particularity of exercise intervention, the blind method of exercise intervention cannot be achieved, which may lead to the risk of bias in the article. (3) There were differences in the design schemes of the included studies, such as exercise, frequency, intensity, and length of intervention, which may lead to heterogeneity in this study. (4) Subgroup analysis was performed only based on the intervention method in the control group, and the effect of more variables on patients with NSLBP should be further explored. (5) There were only three included articles with the McKenzie method as the control group. Conclusions drawn from a small number of studies may be biased, and more studies need to be included to compare the effect of MCT with the McKenzie method in the future.

### Recommendations for future studies

Future research on exercise treatment for NSLBP should evaluate other relevant patient outcomes (e.g. muscle thickness, muscle activation mode) aligned with the proposed mechanisms of exercise treatment, as this will guide individuals and clinicians in their choice for the best treatment. Additionally, examining intervention characteristics (e.g. frequency, intensity, and length) and their relationship with effectiveness of exercise with individual participant data was beyond the scope of this review. This would be important to consider in future research.

## Conclusions

The results show that MCT can effectively relieve pain and improve physical function in patients with NSLBP. Specifically, MCT is superior to PT in alleviating pain and improving physical function, but not different from Pilates and the McKenzie method. Therefore, MCT, Pilates, and the McKenzie method should be encouraged as exercise interventions for NSLBP rehabilitation.

### Supplementary Information

**Additional file 1. Table 1**: GRADE certainty grading evaluation.

## Data Availability

The authors declare that all data supporting the findings of this study are available within the article (and/or OSF: osf.io/me2sq).
