# Supplementary material for: How to tackle non-specific low back pain among adult patients? A systematic review with a meta-analysis to compare four interventions
Source: J Orthop Surg Res. 2024 Jan 3;19:1. doi: 10.1186/s13018-023-04392-2 (PMC10763207; doi:10.1186/s13018-023-04392-2)
Supplement: Supplementary file 1 — Additional file 1. Table 1: GRADE certainty grading evaluation. [file 13018_2023_4392_MOESM1_ESM.docx]

**Supplementary material Table 1** GRADE certainty grading evaluation.

| **Certainty assessment** | | | | | | | **№ of patients** | | **Effect** | | **Certainty** | **Importance** |
| --- | --- | --- | --- | --- | --- | --- | --- | --- | --- | --- | --- | --- |
| **№ of studies** | **Study design** | **Risk of bias** | **Inconsistency** | **Indirectness** | **Imprecision** | **Other considerations** | **MCT** | **other three interventions** | **Relative (95% CI)** | **Absolute (95% CI)** |  |  |
| **Pain** | | | | | | | | | | | | |
| 24 | randomised trials | not serious | serious | not serious | not serious | none | 571 | 574 | - | SMD **0.65 lower** (1 lower to 0.29 lower) | ⨁⨁⨁◯ Moderate | critical |
| **Pain - Pilate** | | | | | | | | | | | | |
| 4 | randomised trials | not serious | serious | not serious | serious | none | 64 | 65 | - | SMD **0.13 higher** (0.56 lower to 0.83 higher) | ⨁⨁◯◯ Low | critical |
| **Pain - Mckenzie** | | | | | | | | | | | | |
| 3 | randomised trials | not serious | serious | not serious | serious | none | 60 | 61 | - | SMD **0.03 lower** (0.75 lower to 0.68 higher) | ⨁⨁◯◯ Low | critical |
| **Pain - Physical therapy** | | | | | | | | | | | | |
| 17 | randomised trials | not serious | serious | not serious | not serious | none | 447 | 448 | - | SMD **0.92 lower** (1.34 lower to 0.5 lower) | ⨁⨁⨁◯ Moderate | critical |
| **Physical function** | | | | | | | | | | | | |
| 23 | randomised trials | not serious | serious | not serious | not serious | none | 544 | 548 | - | SMD **0.76 lower** (1.22 lower to 0.31 lower) | ⨁⨁⨁◯ Moderate | critical |
| **Physical function - Pilate** | | | | | | | | | | | | |
| 4 | randomised trials | not serious | serious | not serious | serious | none | 64 | 65 | - | SMD **0.1 higher** (0.72 lower to 0.91 higher) | ⨁⨁◯◯ Low | critical |
| **Physical function - Mckenzie** | | | | | | | | | | | | |
| 3 | randomised trials | not serious | serious | not serious | serious | none | 60 | 61 | - | SMD **0.03 lower** (1 lower to 0.94 higher) | ⨁⨁◯◯ Low | critical |
| **Physical function - Physical therapy** | | | | | | | | | | | | |
| 16 | randomised trials | not serious | serious | not serious | not serious | none | 420 | 422 | - | SMD **1.15 lower** (1.72 lower to 0.57 lower) | ⨁⨁⨁◯ Moderate | critical |

***Note.*** CI: confidence interval; SMD: standardised mean difference
